# Protective effects of bilberry and lingonberry extracts against blue light-emitting diode light-induced retinal photoreceptor cell damage *in vitro*

**DOI:** 10.1186/1472-6882-14-120

**Published:** 2014-04-02

**Authors:** Kenjirou Ogawa, Yoshiki Kuse, Kazuhiro Tsuruma, Saori Kobayashi, Masamitsu Shimazawa, Hideaki Hara

**Affiliations:** 1 Molecular Pharmacology, Department of Biofunctional Evaluation, Gifu Pharmaceutical University, 1-25-4 Daigaku-nishi, Gifu 501-1196, Japan; 2 Wakasa Seikatsu Co. Ltd., 22 Naginataboko-cho, Shijo-Karasuma, Shimogyo-ku, Kyoto 600-8008, Japan

**Keywords:** Anthocyanin, Bilberry, Blue LED light, Lingonberry, Proanthocyanidin, Resveratrol, Retinal photoreceptor

## Abstract

**Background:**

Blue light is a high-energy or short-wavelength visible light, which induces retinal diseases such as age-related macular degeneration and retinitis pigmentosa. Bilberry (*Vaccinium myrtillus* L.) and lingonberry (*Vaccinium vitis-idaea*) contain high amounts of polyphenols (anthocyanins, resveratrol, and proanthocyanidins) and thus confer health benefits. This study aimed to determine the protective effects and mechanism of action of bilberry extract (B-ext) and lingonberry extract (L-ext) and their active components against blue light-emitting diode (LED) light-induced retinal photoreceptor cell damage.

**Methods:**

Cultured murine photoreceptor (661 W) cells were exposed to blue LED light following treatment with B-ext, L-ext, or their constituents (cyanidin, delphinidin, malvidin, *trans*-resveratrol, and procyanidin B2). 661 W cell viability was assessed using a tetrazolium salt (WST-8) assay and Hoechst 33342 nuclear staining, and intracellular reactive oxygen species (ROS) production was determined using CM-H_2_DCFDA after blue LED light exposure. Activation of p38 mitogen-activated protein kinase (p38 MAPK), nuclear factor-kappa B (NF-κB), and LC3, an ubiquitin-like protein that is necessary for the formation of autophagosomes, were analyzed using Western blotting. Caspase-3/7 activation caused by blue LED light exposure in 661 W cells was determined using a caspase-3/7 assay kit.

**Results:**

B-ext, L-ext, NAC, and their active components improved the viability of 661 W cells and inhibited the generation of intracellular ROS induced by blue LED light irradiation. Furthermore, B-ext and L-ext inhibited the activation of p38 MAPK and NF-κB induced by blue LED light exposure. Finally, B-ext, L-ext, and NAC inhibited caspase-3/7 activation and autophagy.

**Conclusions:**

These findings suggest that B-ext and L-ext containing high amounts of polyphenols exert protective effects against blue LED light-induced retinal photoreceptor cell damage mainly through inhibition of ROS production and activation of pro-apoptotic proteins.

## Background

High-energy visible light has a wavelength in the range of 380 to 530 nm and is present in sunlight, fluorescent light, and light-emitting diode (LED) light. Blue light (from 450 to 495 nm) is high-energy visible light and is related to the pathogenesis of age-related macular degeneration and retinitis pigmentosa [1,2]. A previous report suggested that retinal damage is inversely proportional to wavelength (from 379 to 559 nm) of light in a rat *in vivo* model [3]. In another previous study using rhesus macaque, retinal dysfunction and damage induced by blue LED light were observed as residual infiltration in retinal pigment endothelial (RPE) cells and the photoreceptor outer segment [4]. Blue light-induced RPE cell damage is caused by the accumulation of lipofuscin, such as *bis*-retinoid, *N*-retinylidene-*N*-retinylethanolamine (A2E), and its photoisomers, which lead to reactive oxygen species (ROS) generation by blue light stimulation in the mitochondria, resulting in membrane lipid peroxidation [5]. RPE cell death induced by blue light is mediated by the activation of caspase-3; therefore, nuclear apoptosis is attenuated by the caspase-3 inhibitor Z-DEVD-fmk [6]. Blue light-induced retinal photoreceptor cell damage is also mediated by caspase-3 [7] and rhodopsin *in vivo*, and the extent of its bleaching and its regeneration and visual transduction proteins determine the degree of damage [8].

Bilberry (*Vaccinium myrtillus* L.) and lingonberry (*Vaccinium vitis-idaea*), members of the Ericaceous family, grow in the forests of northern Europe. Bilberry contains 15 different anthocyanins, including 5 anthocyanidins (delphinidin, cyanidin, malvidin, petunidin, and peonidin), and 3 sugars (glucose, galactose, and arabinose). The bilberry extract (B-ext) has potent antioxidant properties [9]; inhibits platelet aggregation [10]; and improves vascular tone, blood flow, and vasoprotection [11,12]. Furthermore, bilberry has been reported to improve visual function in animal models and clinical trials [13,14]. Animal studies have demonstrated B-ext to be beneficial in preventing retinal inflammation and cataracts [15]. Our previous studies have shown a neuroprotective effect against retinal neuronal damage induced by *N*-methyl-d-aspartic acid in mice [16] and an inhibitory effect against angiogenesis in a mouse model of oxygen-induced retinopathy [17].Lingonberry is used in traditional medicine for the treatment of frequent urination, sore eyes, toothache, snow blindness, and thrush [18].

Lingonberry extract (L-ext) contains high amounts of phenolic antioxidants (*trans*-resveratrol and proanthocyanidin). We previously reported that B-ext, L-ext, and their active components (delphinidin, cyanidin, malvidin, resveratrol, procyanidin) protected against retinal photoreceptor cell damage induced by ultraviolet A light (wavelength of 365 nm) exposure [19]. However, the blue light has a high permeability, higher damage, and risk to eye than ultraviolet light, and preventing blue light-induced damage is very important. Thus, the present study aimed to investigate the effects of B-ext, L-ext and their active components, and to elucidate their mechanism of action against blue light (high specificity blue light by LED)-induced retinal photoreceptor cell damage *in vitro*. This study demonstrated that bilberry and lingonberry containing anthocyanidins, procyanidin, and resveratrol exert protective effects against blue LED light-induced retinal photoreceptor cell damage by regulating the activation of NF-κB, p38 MAPK, autophagy, and caspase-3/7 mainly through suppression of ROS generation.

## Methods

### Materials

B-ext (bilberry ethanol extract containing anthocyanins) and L-ext (lingonberry ethanol extract containing *trans*-resveratrol and proanthocyanidins) were purchased from Beijing Gingko Group Japan Co. Ltd. (Tokyo, Japan), and their compositions were confirmed by high performance liquid chromatography (HPLC). Previously, the active components of both extracts were analyzed by HPLC and UV-visible absorption spectroscopy with each standard preparation [19]. B-ext contained 38.4% anthocyanins, 14.1% delphinidin, 9.1% cyanidin, and 6.1% malvidin. L-ext contained 10.6% *trans*-resveratrol and 43.0% proanthocyanidin. The rest of the composition in both extracts was mostly carbohydrates. Delphinidin, cyanidin, malvidin, and procyanidin B2 were purchased from Extrasynthese (Genay Cedex, France). *trans*-Resveratrol was purchased from Tokyo Chemical Industry Co. Ltd. (Tokyo, Japan). *N*-Acetyl-l-cysteine (NAC), a positive control antioxidant, was purchased from Sigma-Aldrich (St Louis, MO, USA). Cell Counting Kit-8 (CCK-8) was purchased from Dojindo Laboratories (Kumamoto, Japan). Hoechst 33342, propidium iodide (PI), and 5-(and-6)-chloromethyl-2,7- dichlorodihydrofluorescein diacetate acetyl ester (CM-H_2_DCFDA) were purchased from Invitrogen (Eugene, OR, USA). Antibodies against phosphorylated p38 MAPK, total p38 MAPK, activated NF-κB, total NF-κB, and LC3 were purchased from Cell Signaling Technology (Beverly, MA, USA). Antibody against β-actin was purchased from Sigma-Aldrich. The Caspase-Glo^®^ 3/7 assay kit was purchased from Promega Co. (Madison, Wisconsin, USA). The 661 W cells were a kind gift from Dr. Muayyad R. Al-Ubaidi (University of Oklahoma Health Sciences Center, Oklahoma City, OK, USA). The blue LED light irradiation devices with 12 blue LED bulbs (wavelength of 460–470 nm) were purchased from M-trust Co. Ltd. (Hyogo, Japan). The LM-332 light meter was purchased from AS ONE Co. Ltd. (Osaka, Japan).

### Cell culture

The 661 W cells were maintained in Dulbecco’s modified Eagle’s medium (DMEM) containing 10% fetal bovine serum (FBS), 100 U/mL penicillin, and 100 μg/mL streptomycin at 37°C in a humidified atmosphere with 5% CO_2_. Cells were passaged by trypsinization every 2–3 d.

### Measurement of cellular metabolic activity after blue LED light exposure

**Figure 1:**
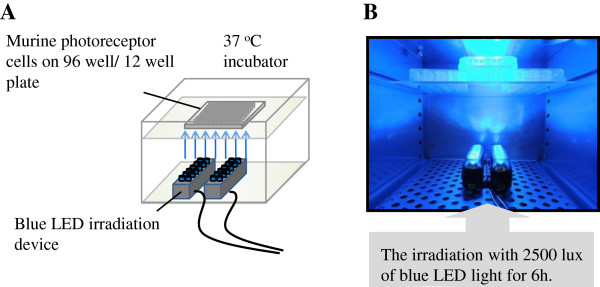
**Model of the blue LED light irradiation-induced murine photoreceptor (661 W) cell damage. (A)** 661 W cells on a 96- or 12-well plate were exposed to blue LED light irradiation at the bottom of a 37°C incubator. **(B)** A magnified view of the Blue LED light irradiation with 2500 lx at the bottom.

661 W cells (3 × 10^3^ cells/100 μL) were seeded onto a 96-well plate and cultured at 37°C for 24 h. We first investigated the effects of NAC, bilberry, lingonberry, and their active components at various concentrations and determined the effective extract concentrations. In this study, we presented the data on specific extract concentrations of each sample against blue LED light-induced photoreceptor cell damage. At 70–80% confluence, the medium was replaced by DMEM containing 1% FBS and was placed at 37°C for 3 h. Then, 1% FBS-DMEM containing 9-*cis*-retinal (at a final concentration of 2.5 μM) was added to all wells. After 4 h of treatment with 9-*cis*-retinal, 1% FBS-DMEM containing B-ext (at final concentrations of 1–10 μg/mL), L-ext (at final concentrations of 1–10 μg/mL), NAC (at final concentrations of 0.3 and 1 μM), anthocyanidins (at final concentrations of 1–10 μM), *trans*-resveratrol (at final concentrations of 1–10 μM), or procyanidin B2 (at final concentrations of 1–10 μM) was added to each well. The cells treated with DMEM containing only 1% FBS and exposed to blue LED light were designated as the vehicle group and used for comparison with the groups treated with each agent. After 1 h of preincubation with agents, the 661 W cells were exposed to 2500 lx of blue LED light at wavelength 460–470 nm for 6 h (Figure 1A and B); the cellular metabolic activity was then immediately measured by using a water-soluble tetrazolium salt,2-(2-methoxy-4-nitrophenyl)-3-(4-nitrophenyl)-5- (2,4-disulfophenyl)-2*H*-tetrazolium monosodium salt (WST-8). Briefly, 10 μL of CCK-8 was added to each well, and the cells were incubated at 37°C for 2 h; the absorbance was measured at 492 nm (reference wavelength, 660 nm) using SkanIt Re for Varioskan Flash 2.4 (ThermoFisher Scientific Inc., Waltham, MA, USA).

### Cell death analysis after blue LED light exposure

After 12 h of blue LED light exposure, Hoechst 33342 (excitation, 360 nm; emission, 490 nm) and PI were added to the culture medium at final concentrations of 8.1 and 1.5 μM, respectively, followed by incubation for 15 min. Micrographs through fluorescence filters for Hoechst 33342 (U-MWU, Olympus Co., Tokyo, Japan) and PI (U-MWIG, Olympus) were acquired using a charge-coupled device camera (DP30BW, Olympus). The number of dead cells was determined.

### Measurement of cellular ROS production after blue light exposure

Intracellular ROS production caused by blue LED light exposure in 661 W cells was determined using CM-H_2_DCFDA. CM-H_2_DCFDA is converted by an intracellular esterase into dichlorodihydrofluorescein (DCFH). Then, the ROS oxidize a non-fluorescent DCFH to a fluorescent DCFH. After blue LED light exposure, CM-H_2_DCFDA was added to the culture medium at a final concentration of 10 μM, followed by incubation at 37°C for 1 h. Fluorescence was then measured using a fluorescence spectrophotometer at 488 nm (excitation) and 525 nm (emission). The number of cells was determined by Hoechst 33342 staining and was used to calculate ROS production per cell [20].

### Western blot analysis

The 661 W cells (3 × 10^4^ cells/mL) were seeded onto a 12-well plate and cultured at 37°C for 24 h. After blue LED light exposure, the 661 W cells were lysed in a cell lysis buffer (RIPA buffer) with phosphatase inhibitor cocktails 2 and 3 (P5726 and P0044, Sigma-Aldrich) and a protease inhibitor (P8340, Sigma-Aldrich). The lysate was centrifuged at 12,000 *g* for 20 min, and the supernatant was collected for analysis. Protein concentration was determined with a BCA protein assay kit (ThermoFisher Scientific Inc.), with bovine serum albumin as standard. An equal volume of protein sample and sample buffer with 10% 2-mercaptoethanol was electrophoresed with a 10% sodium dodecyl sulfate-polyacrylamide gel, and the separated proteins were then transferred onto a polyvinylidene difluoride membrane (Immobilon-P, Millipore Corporation, Billerica, MA, USA). The membrane was immunoblotted with the following primary antibodies: rabbit anti-phospho-p38 MAPK, rabbit anti-p38 MAPK, rabbit anti-activated NF-κB, rabbit anti-NF-κB, and rabbit anti-LC3 (1 : 1000; Cell Signaling Technology), and mouse anti-β-actin (1 : 5000; Sigma-Aldrich)*. HRP-conjugated goat anti-rabbit or goat anti-mouse secondary antibody was used (1:2000; ThermoFisher Scientific Inc.). Immunoreactive bands were visualized using a chemiluminescent substrate (ImmunoStar^®^LD, Wako-Junyaku Inc., Osaka, Japan). Band densities were measured using an imaging analyzer (LAS-4000 mini, Fujifilm, Tokyo, Japan), a gel analysis software (Image Reader LAS-4000, Fujifilm), and a detected band analysis software (Malti Gauge, Fujifilm).

### Measurement of caspase-3/7 activity after blue light exposure

Caspase-3/7 activation caused by blue LED light exposure in 661 W cells was determined using a caspase-3/7 assay kit. After 12 h of blue LED light exposure, Caspase-Glo^®^ 3/7 reagent was added to a 96-well plate, which was incubated at 37°C for 1 h. The 96-well plate was placed in a plate holder in a fluorescence spectrophotometer, and luminescence and fluorescence were measured. The number of cells was determined by Hoechst 33342 staining and used to calculate caspase-3/7 activity per cell.

### Statistical analysis

Data are presented as means ± SEM. Statistical comparisons were made using one-way analysis of variance followed by Student’s *t*-test or Dunnett’s multiple comparison test. A value of *p* < 0.05 was considered statistically significant.

## Results

### Inhibitory effects of B-ext and L-ext on photoreceptor cellular morphological alterations and metabolic activity reduction after blue LED light exposure

**Figure 2:**
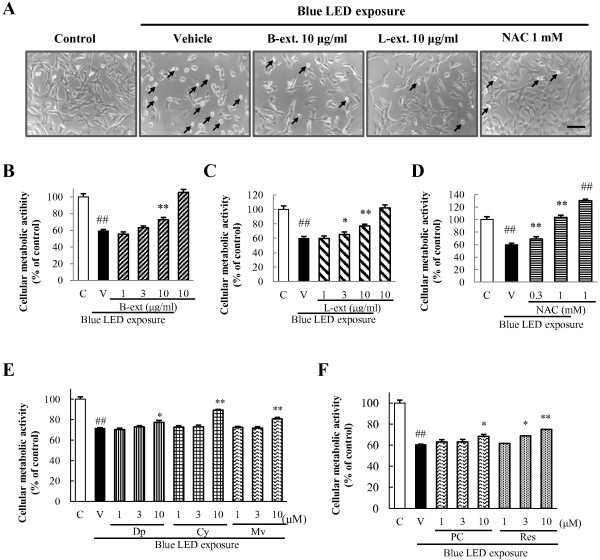
**B-ext, L-ext, their active components, and NAC improved metabolic activity reduction in 661 W cells. (A)** Cytomorphology of 661 W cells after 24 h of blue LED light exposure. Small arrows indicate apoptotic cells. Scale bar, 50 μm. Inhibitory effects of B-ext **(B)**, L-ext **(C)**, NAC **(D)**, delphinidin, cyanidin, malvidin **(E)**, resveratrol, and procyanidin B2 **(F)** on blue LED light-induced reduction of metabolic activity in 661 W cells. Metabolic activity was assessed by incubating the cells with CCK-8 reagent for 2 h at 37°C; photometric data were obtained at 492/660 nm. Cells were treated with B-ext, L-ext, NAC, delphinidin, cyanidin, malvidin, *trans-*resveratrol, and procyanidin B2 for 1 h, and then exposed to 2500 lx of blue LED light for 6 h. Data are means ± SEM (*n* = 6). C, control; V, vehicle; B-ext, bilberry extract; L-ext, lingonberry extract; NAC, *N*-acetyl-l-cysteine; Dp, delphinidin; Cy, cyanidin; Mv, malvidin; PC, procyanidin B2; Res, *trans*-resveratrol. ^##^*p* < 0.01 vs. control; *p < 0.05, **p < 0.01 vs. the vehicle-treated group (Dunnett’s multiple comparison test or Student’s *t*-test).

We first established the blue LED light-induced murine photoreceptor (661 W) cell damage model *in vitro* (Figure 1). Then, we investigated the effects of B-ext, L-ext, and NAC, a positive control antioxidant, at various concentrations, and we determined the effective concentration of each agent. We analyzed the effects of B-ext, L-ext, and NAC on the morphological alterations induced by blue LED light in the 661 W cell cultures. The adherence to 96-well plate bottom of vehicle-treated photoreceptor cells were attenuated and the cytomorphology of that deformed spherical, which indicated apoptotic cells, after blue LED light exposure. However, pretreatment with 10 μg/mL B-ext, 10 μg/mL L-ext, or 1 mM NAC prevented the apoptotic morphological defects by blue LED light exposure (Figure 2A). Treatment with 10 μg/mL B-ext, 3 and 10 μg/mL L-ext, or 0.3 and 1 mM NAC significantly improved the reduction in metabolic activity of 661 W cells induced by blue LED light exposure (Figure 2B–D). Furthermore, B-ext and L-ext active components—delphinidin (10 μM), cyanidin (10 μM), malvidin (10 μM), procyanidin B2 (10 μM), and *trans*-resveratrol (3 and 10 μM)—significantly improved the metabolic activity of 661 W cells after blue LED light exposure (Figure 2E and F).

### Inhibitory effects of B-ext and L-ext and their active components on blue LED light-induced photoreceptor cell death

**Figure 3:**
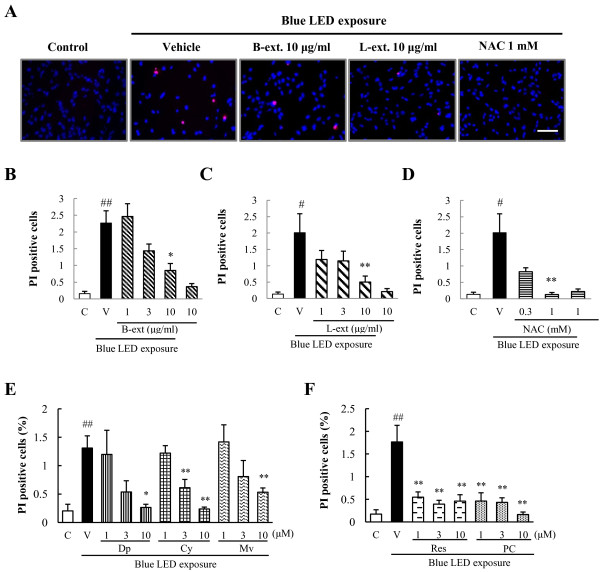
**B-ext, L-ext, their active components, and NAC inhibited blue LED light-induced 661 W cell death. (A)** Representative fluorescence microscopy images of Hoechst 33342 (blue color) and propidium iodide (PI) (red color) staining after 12 h of blue LED light exposure. Scale bar, 50 μm. Inhibitory effects of B-ext **(B)**, L-ext **(C)**, NAC **(D)**, delphinidin, cyanidin, malvidin **(E)**, resveratrol, and procyanidin B2 **(F)** on blue LED light-induced retinal cell death in 661 W cells. The number of cells exhibiting PI fluorescence was counted and expressed as a percentage of Hoechst 33342-positive cells. Cells were treated with B-ext, L-ext, NAC, delphinidin, cyanidin, malvidin, *trans-*resveratrol, and procyanidin B2 for 1 h, and then exposed to 2500 lx of blue LED light for 6 h. Data are means ± SEM (*n* = 6). C, control; V, vehicle; B-ext, bilberry extract; L-ext, lingonberry extract; NAC, *N*-acetyl-l-cysteine; Dp, delphinidin; Cy, cyanidin; Mv, malvidin; Res, *trans*-resveratrol; PC, procyanidin B2. ^##^*p* < 0.01 vs. control; *p < 0.05, **p < 0.01 vs. the vehicle-treated group (Dunnett’s multiple comparison test or Student’s *t*-test).

We counted the number of blue LED light-induced dead photoreceptor cells exhibiting PI fluorescence, expressed as a percentage of cells exhibiting Hoechst 33342 fluorescence to investigate the protective effects of B-ext, L-ext, and their active components at various concentrations against blue LED light-induced cell death in 661 W cells. Representative images of Hoechst 33342 and PI staining shown in Figure 3A. Treatment with 1 mM NAC significantly inhibited 661 W cell death induced by blue LED light exposure (Figure 3D). Treatment with 10 μg/mL B-ext, 10 μg/mL L-ext, B-ext active components (10 μM delphinidin, 3 and 10 μM cyanidin, and 10 μM malvidin), and those of L-ext (1–10 μM *trans*-resveratrol and 1–10 μM procyanidin B2) significantly inhibited 661 W cell death in a concentration-dependent manner (Figure 3B, C, E, F).

### Inhibitory effects of B-ext and L-ext against blue light-induced ROS production in retinal photoreceptor cell cultures

**Figure 4:**
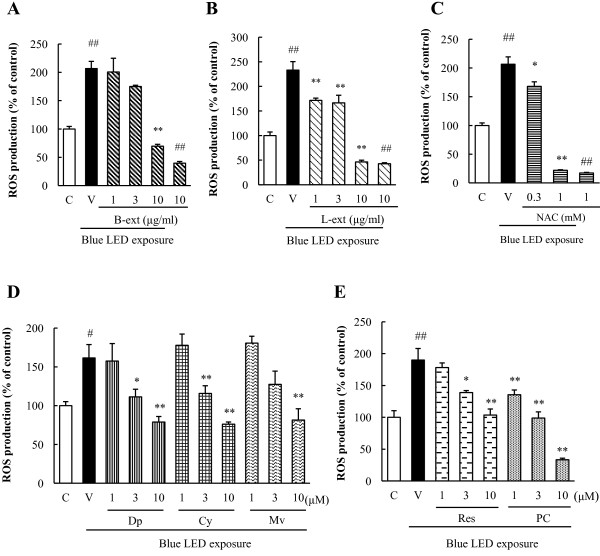
**B-ext, L-ext, their components, and NAC inhibited production of ROS in 661 W cells**. The cells were pretreated with B-ext, L-ext, delphinidin, cyanidin, malvidin, *trans*-resveratrol, and procyanidin B2 for 1 h, and then exposed to 2500 lx of blue LED light for 6 h. B-ext **(A)**, L-ext **(B)**, NAC **(C)**, delphinidin, cyanidin, malvidin **(D)**, *trans*-resveratrol, and procyanidin B2 **(E)** inhibited ROS production in cells induced by blue LED light exposure in a concentration-dependent manner. Intracellular ROS levels were determined by measuring the fluorescence of CM-H2DCFDA (excitation, 488 nm; emission, 525 nm) after blue LED light exposure for 1 h. Data are represented as means ± SEM (*n* = 6). C, control; V, vehicle; B-ext, bilberry extract; L-ext, lingonberry extract; Dp, delphinidin; Cy, cyanidin; Mv, malvidin; Res, *trans-*resveratrol; PC, procyanidin B2. ^##^*p* < 0.01 vs. control; *p < 0.05, **p < 0.01 vs. the vehicle-treated group (Dunnett’s multiple comparison test or Student’s *t*-test).

Intercellular ROS production, which is converted to a fluorescent product (CM-H_2_DCF) upon exposure to ROS, was increased by blue LED light exposure, and 0.3 and 1 mM NAC significantly reduced the blue LED light-induced ROS production in 661 W cells (Figure 4C). Treatment with 10 μg/mL B-ext, 1–10 μg/mL L-ext, and the active components of B-ext and L-ext—delphinidin (3 and 10 μM), cyanidin (3 and 10 μM), malvidin (10 μM), *trans-*resveratrol (3 and 10 μM), and procyanidin B2 (1–10 μM)—significantly reduced the blue LED light-induced ROS production in 661 W cells in a concentration-dependent manner (Figure 4A, B, D, E).

### Regulatory effects of B-ext and L-ext on the phosphorylation of stress response proteins in photoreceptor cells after blue light exposure

**Figure 5:**
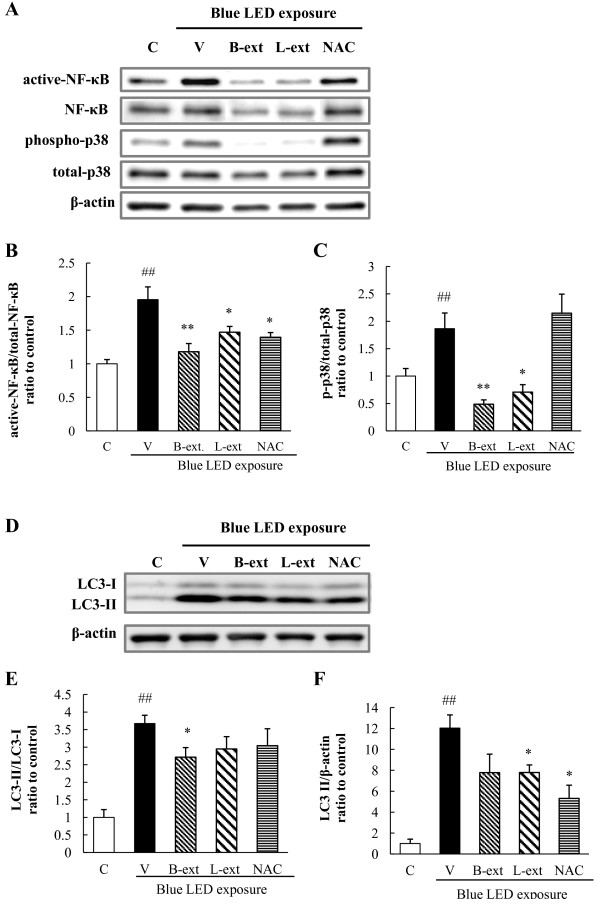
**The effects of B-ext, L-ext, and NAC on Western blot analysis.** Western blots showing the effects of B-ext, L-ext, and NAC on blue LED light-induced activation of NF-κB, phosphorylation of p38 MAPK, and conversion of LC3-I to LC3-II in 661 W cells. Cells were pretreated with B-ext, L-ext, or NAC for 1 h, and then exposed to 2500 lx of blue LED light for 3 h. Then, the cells were collected, and lysates were analyzed by western blotting. **(A)** Representative band images show immunoreactivities against activated NF-κB, NF-κB, phospho-p38, and total p38. **(B and C)** Quantitative analysis of band densities. **(D)** Representative band images show immunoreactivities against LC3-I and LC3-II. **(E and F)** Quantitative analysis of band densities. Data are represented as means ± SEM (*n* = 5–6). C, control; V, vehicle; B-ext, bilberry extract; L-ext, lingonberry extract. ^##^*p* < 0.01 vs. control; *p < 0.05, **p < 0.01 vs. the vehicle-treated group (Student’s *t*-test).

We performed Western blot analysis to investigate the regulatory effects of B-ext, L-ext, and NAC on the p38 MAPK stress response pathway, NF-κB, and LC3 autophagy signaling after exposure to blue LED light. Blue LED light exposure increased the phosphorylation of p38 MAPK and NF-κB activation, and treatments with 10 μg/mL B-ext and 10 μg/mL L-ext significantly inhibited the activation of p38 MAPK and NF-κB in 661 W cells (Figure 5A–C). Furthermore, blue LED light-induced upregulation of LC3 or conversion from LC3-I to LC3-II and the formation of autophagosomes induced by autophagy were inhibited by treatment with B-ext, L-ext, and NAC (Figure 5D–F).

### Inhibitory effects of B-ext and L-ext on the activation of caspase-3/7 in photoreceptor cells after blue light exposure

**Figure 6:**
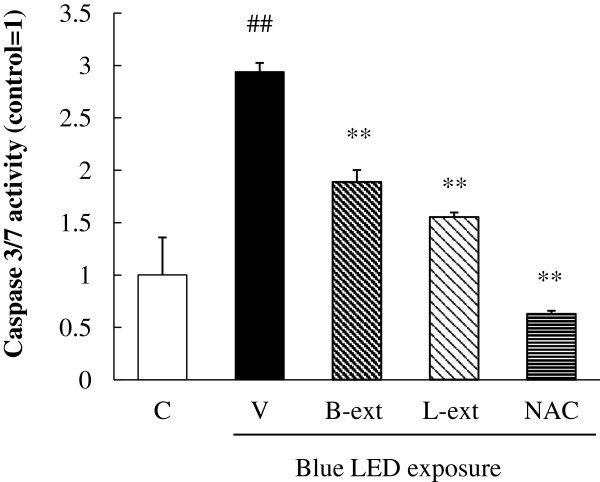
**Effects of B-ext, L-ext, and NAC on caspase 3/7 activation in 661 W cells.** Cells were pretreated with B-ext, L-ext, or NAC for 1 h, and then exposed to 2500 lx of blue LED light for 6 h. The luminescence and fluorescence of the cells cultured in a 96-well plate were measured with a spectrophotometer, and the caspase-3/7 activity per cell was calculated. Data are represented as means ± SEM (*n* = 6). C, control; V, vehicle; B-ext, bilberry extract; L-ext, lingonberry extract. ^##^*p* < 0.01 vs. control; **p < 0.01 vs. the vehicle-treated group (Student’s *t*-test).

We analyzed caspase-3/7 activation using a caspase-3/7 assay kit after 12 h of blue LED light exposure. We measured the luminescence and fluorescence of cells cultured in a 96-well plate using a spectrophotometer and then calculated the caspase-3/7 activity per cell. Caspase-3/7 activity was increased by blue LED light exposure, and treatments with 10 μg/mL B-ext, 10 μg/mL L-ext, and 1 mM NAC significantly inhibited the activation of caspase-3/7 (Figure 6).

## Discussion

In this study, we focused on the protective effects of B-ext, L-ext, and their active components against retinal injury caused by exposure to blue LED light (a part of the high-energy visible light) in photoreceptor cells. We first examined the effects of B-ext, L-ext, and their active components of both extracts against blue LED light-induced photoreceptor cell damage and ROS generation. We determined the effective concentration of B-ext to be 10 μg/mL, which contained 1.41 μg/mL (4.65 μM) delphinidin, 0.91 μg/mL (3.17 μM) cyanidin, and 0.61 μg/mL (1.84 μM) malvidin, and that of L-ext to be 10 μg/mL, which contained 1.06 μg/mL (4.64 μM) *trans*-resveratrol and 4.30 μg/mL (7.43 μM) procyanidins (Figures 2, 3 and 4). These findings suggest that the active components cyanidin, *trans*-resveratrol, and procyanidins, at concentrations of 3–10 μM, 3–10 μM, and 1–10 μM, respectively, are highly effective in inhibiting blue LED light-induced cell death through ROS generation (Figures 3 and 4). B-ext contains 15 different anthocyanins, however, we could not purchase B-ext containing all anthocyanins in glycosidic form. In the results, the main anthocyanidins exerted protective effects (Figures 2, 3 and 4). We then examined cyanidin-3-glucoside, a glycosidic anthocyanin found in B-ext, and the results were similar to those obtained for cyanidin (data not shown). Thus, future studies should investigate the protective effects of other glycosidic anthocyanins in B-ext, which are absorbed into the bloodstream in the glycosylated form and metabolized in the liver and then transported as anthocyanin and anthocyanin metabolites to the eye [21,22].

Blue light causes damage to mitochondrial DNA and induces free radical production in retinal cells, as previously reported [23]. Free radicals induce lipid peroxidation, protein degeneration, and induction of apoptosis in cells. In this study, we investigated B-ext, L-ext, their active components, and NAC, and found that they exert protective effects against blue LED light-induced photoreceptor cell damage (Figures 3 and 4). We previously reported that B-ext and its anthocyanidins scavenge superoxide anion radicals and hydroxyl radicals [24]. Furthermore, *trans*-resveratrol and procyanidin have also been found to have free radical-scavenging activity [25,26]. In addition, anthocyanins, procyanidin B2, and *trans*-resveratrol enhance the antioxidant properties of intracellular glutathione and endogenous superoxide dismutase [27-29]. Thus, as shown in this study, B-ext and L-ext may not only scavenge ROS but also improve the cellular ROS scavenging activity in retinal photoreceptor cells.

B-ext and L-ext containing the active components exert not only antioxidant effects but also inhibitory effects against stress response proteins induced by blue LED light exposure. p38 MAPK participates in cellular responses to mitogenic stimuli, including oxidative stress, UV exposure, and light exposure, during cell differentiation and apoptosis. The activation of p38 MAPK by light exposure induces the apoptotic transcription factor activator protein-1 (AP-1) in 661 W cells [30]. A previous report suggested that activation of p38 MAPK occurs because of the generation of singlet oxygen by blue light in the retina [31]. Anthocyanins, *trans*-resveratrol, and procyanidin B2 have antioxidant effects against ROS involving singlet oxygen [32-34]; however, NAC reacts with hydrogen peroxide, hydroxyl radical, and superoxide anion radical, but not singlet oxygen [35]. Kwon et al. [36] and Lim et al. [37] have proposed that delphinidin and cyanidin inhibit phosphorylation of MKK4 and MAPK kinase activation by binding to MKK4 in an ATP-competitive manner. Thus, B-ext containing delphinidin and cyanidin might also directly inhibit the blue LED light-stimulated activation of p38 MAPK.

Light exposure causes oxidative stress through NF-κB activation, which is related to inflammation, cancer, and cell apoptosis [38,39]. A previous *in vivo* study suggested that NF-κB colocalizes with TUNEL-positive cells in mouse retinal photoreceptors after light stimulation and causes light-induced retinal photoreceptor degeneration via the NF-κB/caspase pathway [40]. On the other hand, autophagy, or type II programmed cell death, involves the degradation of long-lived proteins in cells [41]. A previous report showed that oxidative stress and light irradiation stimulate autophagy in photoreceptor cells [42]; in addition, 3-methyladenine, an inhibitor of autophagy, prevents photoreceptor cell death induced by activated caspase-3 with H_2_O_2_ treatment [42]. In this study, the large amount of ROS generated by blue LED light stimulation and the subsequent autophagy activation in photoreceptor cells might contribute, at least in part, to the blue LED light-induced photoreceptor cell death. Furthermore, a previous report suggested that activated caspase-3, -7, and -8 play a role as pro-autophagic agents [43]. Caspase-3/7 play an essential role in photoreceptor cell apoptosis [7] and are activated by stimulation of oxidative stress, endoplasmic reticulum stress [44], and p38 MAPK phosphorylation [45]. In the present study, B-ext and L-ext containing polyphenols might inhibit the activation of NF-κB, autophagy (as the upregulation of LC3-II), and caspase-3/7 mainly through suppression of ROS generation induced by blue LED light exposure (Figures 5 and 6). In addition, NAC might inhibit the activation of caspase-3/7 inducing cell death through scavenging ROS except for singlet oxygen. Finally, we investigated the effects of combination with both B-ext and L-ext. Although we found additive effects of both extracts (data not shown), the difference of action mechanisms in between B-ext and L-ext was not shown except for inhibiting ROS generation.

The metabolism of orally administered anthocyanins, resveratrol, and procyanidins in animals and humans has been reported previously. In a previous human study, plasma concentrations of anthocyanins ranged between 0.56 and 4.46 nmol/L after consumption of cranberry juice containing 94.47 mg of anthocyanins in 15 participants [21]. On the other hand, in a previous study using pigs orally administered with blueberry powder, anthocyanins have been detected in the liver, brain, and eyes [46]. Another previous *in vivo* study using murine ocular inflammation model demonstrated that oral administration of B-ext at 500 mg/kg body weight for 4 d prevented inflammatory retinal damage and visual function in mice [14]. Although the plasma concentration of anthocyanins after oral administration may be lower than the effective concentrations *in vitro* in the present study, anthocyanins may be able to reach the ocular tissues and may have potential eye health benefits. Several studies have shown the biokinetics of resveratrol and procyanidin in humans [47,48], and the plasma concentrations were approximately the effective doses of both resveratrol and procyanidin used in our study. In further research, to determine the metabolism of those components in the eye and investigate the protective effects against blue LED light-induced photoreceptor damage *in vivo* would be necessary in the case of B-ext, L-ext, and those active components.

## Conclusion

In conclusion, we have demonstrated that bilberry and lingonberry containing anthocyanidins, procyanidin, and resveratrol exert protective effects against blue LED light-induced retinal photoreceptor cell damage by regulating the activation of NF-κB, p38 MAPK, autophagy, and caspase-3/7 mainly through suppression of ROS generation.

## Abbreviations

B-ext: Bilberry extract; CM-H2DCFDA: 5-(and )-chloromethyl-2,7- dichlorodihydrofluorescein diacetate acetyl ester; DCFH: Dichlorodihydrofluorescein; JNK: c-Jun N-terminal kinases; LED: L-ext lingonberry extract; MAPK: Mitogen-activated protein kinase; NAC: *N*-Acetyl-l-cysteine; NF-κB: Nuclear factor-kappa B; PI: Propidium iodide; ROS: Reactive oxygen species; WST-8: 2-(2-methoxy-4-nitrophenyl)- 3-(4-nitrophenyl)-5-(2,4-disulfophenyl)-2*H*-tetrazolium monosodium salt.

## Competing interests

The authors declared that they have no competing interests.

## Authors’ contributions

KO designed the study, performed the tests, analyzed the data obtained and drafted this paper. YK and KT supported for design of the study and performance of the tests. SK provided the bilberry and lingonberry extracts. MS and HH supervised the execution of the study. All authors contributed to the manuscript preparation and approved of the final paper.

## Pre-publication history

The pre-publication history for this paper can be accessed here:

http://www.biomedcentral.com/1472-6882/14/120/prepub
